# Self-reported Snoring and Carotid Atherosclerosis in Middle-aged and Older Adults: The Korean Multi-Rural Communities Cohort Study

**DOI:** 10.2188/jea.JE20130114

**Published:** 2014-07-05

**Authors:** Young-Hoon Lee, Sun-Seog Kweon, Bo Youl Choi, Mi Kyung Kim, Byung-Yeol Chun, Dong Hoon Shin, Min-Ho Shin

**Affiliations:** 1 Department of Preventive Medicine & Institute of Wonkwang Medical Science, Wonkwang University School of Medicine, Iksan, Jeonbuk, South Korea; 2 Regional Cardiocerebrovascular Center, Wonkwang University Hospital, Iksan, Jeonbuk, South Korea; 3 Department of Preventive Medicine, Chonnam National University Medical School, Gwangju, South Korea; 4 Jeonnam Regional Cancer Center, Chonnam National University Hwasun Hospital, Hwasun, South Korea; 5 Department of Preventive Medicine, College of Medicine, Hanyang University, Seoul, South Korea; 6 Department of Preventive Medicine, School of Medicine, and Health Promotion Research Center, Kyungpook National University, Daegu, South Korea; 7 Department of Preventive Medicine, Keimyung University School of Medicine, Daegu, South Korea

**Keywords:** snoring, atherosclerosis, carotid intima-media thickness, carotid artery plaque

## Abstract

**Background:**

We investigated the relation of self-reported snoring with carotid intima-media thickness (IMT) and plaque in community-dwelling middle-aged and older adults.

**Methods:**

In total, 7330 community-dwelling subjects in the Korean Multi-Rural Communities Cohort Study were included in the analysis. Common carotid artery IMT (CCA-IMT) and plaque were evaluated by high-resolution B-mode ultrasonography. Snoring status was evaluated using a structured interview.

**Results:**

Snorers had a significantly greater average CCA-IMT than non-snorers (0.726 vs 0.713 mm; *P* < 0.001), after adjusting for age and gender. The odds ratios (OR) for high CCA-IMT (fifth quintile) were significantly higher for snorers than for non-snorers in multivariate-adjusted analysis (OR 1.25, 95% confidence interval [CI] 1.10–1.42). However, there was no significant relationship between snoring and carotid plaques.

**Conclusions:**

Our data suggest that self-reported snoring is significantly associated with increased IMT, but not with the presence of plaques. These findings suggest that early screening and intervention for snoring in the general population are needed to prevent adverse cardiovascular events.

## INTRODUCTION

Snoring is a sound emitted from the upper airway of the throat during sleep and is an indicator of increased resistance through the airway.^1^ Previous studies have reported that male gender, age, obesity, alcohol consumption, cigarette smoking, menopause, physical inactivity, and family history were risk factors for snoring.^2^^–^^9^ Epidemiologic evidence has also shown that self-reported snoring was associated with increased incidence of cardiovascular events.^10^^–^^13^

Since previous epidemiologic studies have suggested that snoring is significantly associated with metabolic syndrome,^14^^,^^15^ metabolic components have been considered as the link between snoring and cardiovascular disease (CVD). Alternatively, a non-metabolic pathway that mediates the relationship between snoring and CVD, such as carotid atherosclerosis, has been proposed.^16^^,^^17^ Increased carotid intima-media thickness (IMT) and atherosclerotic plaques are surrogate markers of subclinical atherosclerosis and strong predictors of CVD, such as stroke and myocardial infarction.^18^^,^^19^ Few epidemiologic studies have examined the association between snoring and carotid atherosclerosis; however, those that did reported inconsistent associations.^20^^,^^21^ To date, whether or not self-reported snoring is significantly associated with carotid atherosclerosis in the general population remains undetermined. The aim of this study was to investigate the association between self-reported snoring and carotid atherosclerosis in community-dwelling middle-aged and elderly Korean men and women.

## METHODS

### Study population

The Korean Multi-Rural Communities Cohort Study, which is part of the Korean Genome Epidemiology Study, was initiated in 2004 with the aim of constructing a genomic cohort and investigating the risk factors for CVD in rural communities. As the baseline, 9696 subjects, aged ≥40 years, were recruited between January 2005 and February 2010 from three centers: Yangpyeong in Gyeonggi-do (*n* = 3183), Namwon in Jeollabuk-do (*n* = 3408), and Goryeong in Gyeongsangbuk-do (*n* = 3105). The majority of the subjects were farmers and housewives. We excluded subjects who had a previous history of coronary heart disease or cerebrovascular disease during the baseline survey, those who did not provide information on their snoring, and those who did not undergo carotid ultrasonography. In total, 7330 subjects (2901 men and 4429 women) aged 40–91 years were included in the final analysis. This study was conducted in accordance with the Declaration of Helsinki guidelines, and all procedures involving human subjects were approved by the Institutional Review Boards of Hanyang University, Chonnam National University, and Keimyung University. All subjects were fully informed of the study content and gave written informed consent for the use of their data.

### Interview and examination

We administered a questionnaire and an examination using a standardized protocol to overcome the limitations of multi-center studies. All interviewers and examiners were trained by the same personnel at the coordinating center. Information on demographics, smoking, alcohol intake, exercise, medical history, and medications was collected, using a questionnaire administered by well-trained interviewers. Height was measured to the nearest 0.1 cm using a standard height scale, and weight in light clothing without shoes was measured to the nearest 0.1 kg using a metric weight scale. Body mass index (BMI) was calculated as weight (kg) divided by height (m) squared. Waist circumference was measured to the nearest 0.1 cm at the midpoint between the lowest rib margin and the iliac crest during expiration.

Blood pressure was measured from the right upper arm, using a standard mercury sphygmomanometer (Baumanometer; WA Baum Co., Inc., Copiague, NY, USA) and a standard cuff. Two consecutive blood-pressure measurements were performed after each subject had been sitting for at least 5 min. The subjects were instructed to relax as much as possible, and not to talk during the measurement procedure. The first appearance (phase I) and disappearance (phase V) of Korotkoff sounds were used to define systolic blood pressure and diastolic blood pressure, respectively. Systolic and diastolic blood pressures were recorded to the nearest 2 mm Hg. If two systolic or diastolic blood pressures were more than 5 mm Hg apart, an additional measurement was performed, and the mean value of the closest two measurements was used for the subsequent analyses.

Blood samples taken from the antecubital vein were collected from each subject during the morning after an 8-h overnight fast, and all biochemical markers were evaluated on the same day. Total serum cholesterol, high-density lipoprotein (HDL) cholesterol, triglyceride, and fasting blood glucose levels were analyzed using an ADVIA1650 Automatic Analyzer (Siemens, New York, NY, USA).

### Information on snoring

Snoring status was evaluated using a structured interview, including two questions: (1) do you know, or have you ever heard that you snore? (yes or no); and (2) how often do you snore? (<1 day per month, 1–3 days per month, 1–3 days per week, 4–5 days per week, ≥6 days per week). Snoring frequency was reclassified into <1 day/week, 1–3 days/week, and ≥4 days/week.

### Carotid ultrasound measurements

The carotid artery was evaluated by using high-resolution B-mode ultrasound equipment (SonoAce-9900; Medison, Seoul, South Korea), equipped with a 7.5-MHz linear array transducer. IMT was determined as the distance from the media-adventitia interface to the intima-lumen interface on the far wall in the longitudinal view. Ultrasonographic images were captured and analyzed by five sonographers at the three centers. A single trained reader at the reading center in the Department of Preventive Medicine at Chonnam National University analyzed the images using Sigma Scan Pro 5.0 (SPSS Inc., Chicago, IL, USA). Between the carotid bulb origin and a point 10 mm proximal to the common carotid artery (CCA), the maximal IMT value in a region free of plaque was determined as the maximal IMT of the left/right CCA. The CCA-IMT, defined as the average of the maximal IMT values of both CCA was used for analysis; ‘high CCA-IMT’ was defined as the fifth quintile of CCA-IMT (≥0.823 mm). The reader also assessed the presence of carotid plaques, which were defined as focal lesions that encroached into the lumen by at least 100% of the surrounding IMT value. The presence of carotid plaques was determined from scans of the CCA and bulb segments. The presence of carotid plaques was recorded if at least one lesion was detected in any segment.

### Statistical analysis

Differences between snorers and non-snorers in subject characteristics were compared using Student’s *t*-test for continuous variables and a chi-square test for categorical variables. Analysis of covariance was used to evaluate IMT differences according to snoring and its frequency after controlling for age and gender. Independent associations of snoring and its frequency with increased IMT and the presence of plaques were analyzed, using multiple logistic regression after adjusting for conventional risk factors. The odds ratios (ORs) are presented with 95% confidence intervals (CIs). A *P*-value of 0.05 was considered to indicate statistical significance. All statistical analyses were performed using the SPSS 15.0 software package (SPSS Inc., Chicago, IL, USA).

## RESULTS

### Baseline characteristics of the study population

The baseline characteristics of the study population according to self-reported snoring status are shown in Table 1. Of the 7330 subjects analyzed, 3462 (47.2%) were snorers and 3868 (52.8%) were non-snorers. The mean age of snorers and non-snorers was 60.0 ± 9.2 and 62.4 ± 10.3 years (*P* < 0.001), respectively. Compared to the non-snorers, snorers had significantly higher BMI, waist circumference, diastolic blood pressure, fasting blood glucose, and triglycerides, as well as lower HDL cholesterol. The prevalences of being male, medication for hypertension and dyslipidemia, current drinking, and regular exercise were significantly higher in snorers than in non-snorers (Table 1). Distributions of self-reported snoring frequency were as follows: 17.4% (19.3% in men, 16.2% in women) reported snoring ≥4 days per week, 14.9% (16.6% in men, 13.8% in women) reported snoring 1–3 days per week, 14.9% (13.5% in men, 15.8% in women) reported snoring <1 day per week, and 52.8% (50.5% in men, 54.3% in women) reported no snoring (data not shown).

**Table 1.** Characteristics of the study population according to self-reported snoring status

	Non-snorers (*n* = 3868)	Snorers (*n* = 3462)	*P*-value
Age, years	62.4 ± 10.3	60.0 ± 9.2	<0.001
Men, *n* (%)	1465 (37.9)	1436 (41.5)	0.002
Body mass index, kg/m^2^	23.5 ± 3.0	25.1 ± 3.2	<0.001
Waist circumference, cm	82.5 ± 8.7	85.9 ± 8.7	<0.001
Systolic blood pressure, mm Hg	124.4 ± 18.3	125.1 ± 16.8	0.065
Diastolic blood pressure, mm Hg	78.0 ± 10.4	79.8 ± 10.2	<0.001
Fasting blood glucose, mg/dL	100.2 ± 26.6	101.9 ± 22.8	0.004
Total cholesterol, mg/dL	198.8 ± 37.0	200.1 ± 36.8	0.168
HDL cholesterol, mg/dL	45.2 ± 10.9	44.3 ± 10.6	<0.001
Total/HDL cholesterol ratio	4.6 ± 1.2	4.7 ± 1.2	<0.001
Triglycerides, mg/dL	122 (88–174)	131 (94–193)	<0.001
Medication for hypertension, *n* (%)	727 (18.8)	861 (24.9)	<0.001
Medication for diabetes, *n* (%)	272 (7.0)	285 (8.2)	0.053
Medication for dyslipidemia, *n* (%)	62 (1.6)	85 (2.5)	0.009
Current smoking, *n* (%)	612 (15.8)	555 (16.0)	0.807
Current drinking, *n* (%)	1619 (41.9)	1667 (48.2)	<0.001
Regular exercise, *n* (%)	1058 (27.4)	1070 (30.9)	0.001
CCA-IMT, mm	0.718 (0.146)	0.714 (0.143)	0.282
Carotid plaque, *n* (%)	1240 (32.1)	1021 (29.5%)	0.018

Data are presented as mean ± standard deviations, or median (interquartile range) or number (percentage).

HDL, high-density lipoprotein; CCA-IMT, common carotid artery intima-media thickness.

No significant differences in CCA-IMT between snorers and non-snorers (0.714 vs 0.718 mm; *P* = 0.282) were identified. However, after adjusting for age and gender, snorers had significantly greater CCA-IMT values than non-snorers (0.726 vs 0.713 mm; *P* < 0.001; data not shown). Snorers had a lower prevalence of carotid plaque than non-snorers in unadjusted analysis (29.5% vs 32.1%; *P* = 0.018) (Table 1).

### Association between snoring and carotid IMT

The relationship between snoring and an increased IMT is shown in Table 2. The ORs for high CCA-IMT (fifth quintile, ≥0.823 mm) among snorers were significantly higher than those among non-snorers in age- and gender-adjusted analysis (OR 1.31, 95% CI 1.16–1.49) and multivariate-adjusted analysis (OR 1.25, 95% CI 1.10–1.42). Compared to non-snorers, the ORs for high CCA-IMT among snorers were significantly higher in subjects who snored 1–3 days/week (age- and gender-adjusted OR 1.40, 95% CI 1.17–1.68; multivariate-adjusted OR 1.36, 95% CI 1.13–1.63) and ≥4 days/week (age- and gender-adjusted OR 1.36, 95% CI 1.16–1.61; multivariate-adjusted OR 1.28, 95% CI 1.08–1.53).

**Table 2.** Relationship between snoring and presence of high CCA-IMT^a^

	Age- and gender-adjusted OR (95% CI)	Multivariate-adjusted^b^ OR (95% CI)
Self-reported snoring status		
Non-snorers (*n* = 3868)	1.00	1.00
Snorers (*n* = 3462)	1.31 (1.16–1.49)	1.25 (1.10–1.42)
Self-reported snoring frequency		
Non-snorers (*n* = 3868)	1.00	1.00
<1 day/week (*n* = 1092)	1.16 (0.96–1.40)	1.11 (0.91–1.34)
1–3 days/week (*n* = 1093)	1.40 (1.17–1.68)	1.36 (1.13–1.63)
≥4 days/week (*n* = 1277)	1.36 (1.16–1.61)	1.28 (1.08–1.53)

CCA-IMT, common carotid artery intima-media thickness.

^a^High CCA-IMT was defined as the fifth quintile (≥0.823 mm).

^b^Adjusted for age, gender, center, body mass index, systolic blood pressure, fasting blood glucose, total to HDL cholesterol ratio, triglycerides, current smoking, excessive alcohol drinking, regular exercise, medication for hypertension, medication for diabetes, and medication for dyslipidemia.

### Association between snoring and carotid plaques

No significant relationship between snoring and carotid plaques was identified in age- and gender-adjusted and multivariate-adjusted analyses. In addition, compared with non-snorers, no significantly higher or lower ORs were identified in subjects who snored <1 day/week, 1–3 days/week, or ≥4 days/week (Table 3).

**Table 3.** Relationship between snoring and presence of carotid plaques

	Age- and gender-adjusted OR (95% CI)	Multivariate-adjusted^a^ OR (95% CI)
Self-reported snoring status		
Non-snorers (*n* = 3868)	1.00	1.00
Snorers (*n* = 3462)	1.06 (0.95–1.18)	1.04 (0.93–1.17)
Self-reported snoring frequency		
Non-snorers (*n* = 3868)	1.00	1.00
<1 day/week (*n* = 1092)	0.94 (0.80–1.10)	0.92 (0.78–1.09)
1–3 days/week (*n* = 1093)	1.12 (0.96–1.31)	1.10 (0.93–1.29)
≥4 days/week (*n* = 1277)	1.10 (0.95–1.27)	1.10 (0.94–1.28)

^a^Adjusted for age, gender, center, body mass index, systolic blood pressure, fasting blood glucose, total to HDL cholesterol ratio, triglycerides, current smoking, excessive alcohol drinking, regular exercise, medication for hypertension, medication for diabetes, and medication for dyslipidemia.

## DISCUSSION

We evaluated the relationship between self-reported snoring and subclinical carotid atherosclerosis in community-dwelling middle-aged and older adults. Our data suggest that, after adjustment for established cardiovascular risk factors, self-reported snoring is significantly associated with increased IMT but not with the presence of plaques.

Sleep-disordered breathing (SDB) is characterized by apnea and hypopnea events during sleep and is a risk factor for cardiovascular outcomes.^22^ Snoring, which is characterized by loud upper airway breathing sounds created by vibration of the pharyngeal walls and associated structures, is regarded as a surrogate marker of SDB.^1^^,^^23^ In meta-analysis, snoring was more frequently reported in men than in women (OR 1.89, 95% CI 1.75–2.03).^2^ The gender difference in the risk of snoring can be mainly explained by the differences in upper airway anatomy.^2^

Most previous studies have focused on the relationship between obstructive sleep apnea (OSA), a common medical condition measured by polysomnography, and subclinical atherosclerosis.^24^^,^^25^ However, compared to OSA, snoring is much more simple to assess via self-reported questionnaire, and this method has been widely used to obtain information, especially in epidemiologic studies.^10^^–^^13^^,^^26^ In addition, self-reported snoring captures symptoms experienced over time, which cannot be achieved by overnight measurement.^20^

Epidemiologic studies have demonstrated that self-reported snoring is significantly associated with future development of CVD.^10^^–^^13^ Several studies have investigated the relationship between self-reported snoring and carotid atherosclerosis, but their findings were not in agreement.^20^^,^^21^ A community-based study of 1050 Chinese subjects found that self-reported snoring was independently associated with increased IMT and the presence of carotid bulb plaque.^20^ Further, the study reported dose-response trends between self-reported snoring frequency and IMT values and the prevalence of plaque.^20^ In contrast, there was no significant association between self-reported snoring and subclinical atherosclerosis, measured by carotid IMT in the Northern Manhattan Study, which included 1605 American subjects.^21^ Another community-based study using polysomnography found no evidence of meaningful associations between SDB, as assessed by the respiratory disturbance index, hypoxemia index, and an arousal index, and carotid IMT or plaque.^27^

Assessment of carotid arteries using B-mode ultrasound imaging is a simple, non-invasive, and cost-effective method for evaluation of carotid atherosclerosis by measuring IMT or by identifying an atherosclerotic plaque. However, increases in IMT and plaque formation are biologically distinct and may reflect different features of atherosclerosis. IMT, which primarily reflects an adaptive hypertrophic response of the media, is regarded as an indicator of early atherosclerosis, whereas plaque, which is a pathological deposit on the intima, primarily represents a later stage of atherosclerosis.^18^^,^^28^^,^^29^

A significant association between the presence of snoring and carotid IMT was identified in our study. More specifically, a significant escalating association between the snoring frequency and CCA-IMT was found. In contrast to a recent community study,^20^ no significant association between snoring and carotid plaques was found in the present study. Differences in the study population and severity of snoring might explain this discrepancy. The absence of an association between snoring and carotid plaques in our study may be due to a number of factors. First, the presence of plaques was evaluated from scans of the carotid artery in common and bulb segments but not in internal segments. No detailed assessments of plaque characteristics, including echogenicity, volume, and height, were performed. The use of the mere presence of plaque as an outcome might have resulted in a non-significant association between snoring and carotid plaques. Second, misclassification of self-reported snoring and its frequency might have occurred due to unawareness or under-reporting of snoring status. Non-differential misclassification might result in underestimation of the true association between snoring on carotid plaques, if such an association existed. Further research is needed to clarify the true association between snoring and carotid plaques.

The pathophysiological mechanisms that link snoring and carotid atherosclerosis remain unclear; however, several mechanisms have been proposed. Hedner et al^30^ proposed that habitual snoring may transmit sufficient vibrational energy through the surrounding tissues to the carotid artery wall. Such vibrations may result in carotid artery endothelial damage, leading to the development of atherosclerosis and the formation and rupture of the plaque. Recent experimental evidence has demonstrated that, during snoring, pressure vibrations occur in the tissues surrounding the carotid artery wall and are transmitted to the carotid artery lumen.^16^^,^^17^ Amplification of these pressure waves across the carotid wall may result in the development of carotid atherosclerosis. Oxidative stress,^31^ sympathetic activation,^32^ inflammation,^33^ platelet aggregation,^34^ and endothelial dysfunction^35^ may also be involved in the mechanism underlying the association of snoring with atherosclerosis and CVD.

Several limitations to the present study should be discussed. First, due to its cross-sectional design, we are unable to make causal inferences between snoring and carotid atherosclerosis. Second, our findings were generated using convenience sampling and should be interpreted with caution when generalizing to a wider population. We were unable to present the response rate or representativeness, because our study population does not represent a target population. Third, our data on snoring status and frequency were obtained from a self-reported questionnaire, with no precise evaluation of sleep disorders or complaints performed using polysomnography. Snoring sounds are commonly described as a nuisance by a bed partner, so a snorer who sleeps alone may be unaware of their snoring status. Misclassification of snoring status due to measurement error might have attenuated the relationship between snoring and carotid atherosclerosis. Despite these limitations, this study represents a valuable contribution to understanding the relationship between snoring and carotid atherosclerosis because of the use of a large community-dwelling population. In addition, this study showed that the snoring-IMT association was positive, even though the population was lean compared to Western populations. Such studies have not been performed as frequently in non-Western populations.

In conclusion, our data suggest that self-reported snoring is independently associated with carotid IMT, but not with carotid plaques. Early screening and intervention for snoring in the general population may be useful to prevent adverse cardiovascular events. Further longitudinal investigations are necessary to clarify the mechanism underlying the association between snoring and carotid atherosclerosis.
